# Effects of physical fitness using a Kinect system for pre-frail community-dwelling older adults

**DOI:** 10.3389/fpubh.2025.1498606

**Published:** 2025-06-11

**Authors:** Wan-Yun Huang, I-Hsiu Liou, Shin-Tsu Chang, Chao-Hsien Lee, Yi-Chun Du, Rong-Ju Cherng

**Affiliations:** ^1^Institute of Allied Health Sciences, College of Medicine, National Cheng Kung University, Tainan, Taiwan; ^2^Department of Physical Medicine and Rehabilitation, Kaohsiung Veterans General Hospital, Kaohsiung, Taiwan; ^3^Department of Physical Medicine and Rehabilitation, National Defense Medical Center, School of Medicine, Tri-Service General Hospital, Taipei, Taiwan; ^4^Department of Social Work, Meiho University, Pingtung, Taiwan; ^5^College of Engineering, Institute of Biomedical Engineering, National Cheng Kung University, Tainan, Taiwan; ^6^Department of Physical Therapy, College of Medicine, National Cheng Kung University, Tainan, Taiwan

**Keywords:** physical fitness, aerobic dance exercise, Kinect system, frailty, older adults

## Abstract

**Purpose:**

The study aimed to examine and compare the effects on physical fitness between aerobic dance exercise using Kinect system and aerobic dance exercise using video in pre-frail community-dwelling older adults.

**Methods:**

The study adopted an assessor-blinded experimental design. Sixty participants enrolled in the study. They were randomly assigned into an experimental group (*n* = 30) and control group (*n* = 30). The experimental group received aerobic dance exercise using Kinect system 30 mins/session, three sessions/week for 8 weeks. The control group received aerobic dance exercise program with video at home. Physical fitness, quality of life and exercise behavior regulation were assessed before, after the intervention (0–8 weeks) and at a 1-month follow-up (0–12 weeks).

**Results:**

Both the experimental and control groups showed improvement in 30 Second Chair Stand Test (30CST) and 6-minute walk tests (6MWT) at 0–8 weeks and at 0–12 weeks. But the experimental group presented significantly more improvement than control group at 0–8 weeks in 30CST and at 0–12 weeks in 6MWT. The experimental group also exhibited significant improvement in Quality-of-Life Questionnaire by World Health Organization scores at 0–8 and 0–12 weeks. A significant difference in Behavior Regulation in Exercise Questionnaire-2 (BREQ-2) scores was also noted in the experimental group at 0–12 weeks (*p* < 0.008).

**Conclusion:**

Both groups of participants improve their physical fitness, but the experimental group improve more in certain measures. Moreover, they also show significant improvement in quality of life, facilitate exercise behavior regulation and improve fitness enthusiasm. Thus, aerobic dance exercise using Kinect system is more effective than traditional training for pre-frail community-dwelling older adults.

**Clinical Trial Registration:**

https://register.clinicaltrials.gov/prs/beta/records, Identifier: NCT06216236.

## 1 Introduction

Frailty is an age-related physiological decline syndrome (1), with weakness being the most common initial manifestation. People between the ages of 40 and 60 begin to lose muscle strength due to the loss of muscle mass and quality (2). The osteoporotic fracture index (SOF index) is one of the tools used to screen for frailty in older adults. The SOF index consists of three indicators: weight loss, lower-extremity function, and reduced energy level. If a subject meets more than two indicators, they will be assessed as “frail,” a subject who meets only one item will be considered as “pre-frail,” and if they meet none of the criteria, they will be considered healthy (3). Studies have shown that effective screening of frail older adults individuals and early intervention with health-promoting activities will help extend the time before the disability stage (4, 5). Previous research suggests that interventions such as aerobic exercise, muscle training, balance training, and stretching exercises can improve and delay frailty in older adults (6).

Aerobic dance courses are popular activities for older adults in the community. Aerobic dance consists of physical activities to consume body calories and promote health under a certain time, intensity, and frequency (7). An aerobic teacher or exercise video will guide participants on the correct posture while performing dance moves (8). Aerobic dance is rhythmic and regular, and the intensity of exercise can be adjusted according to the rhythm of the music (9). These exercises consist of various dance moves with music to increase enjoyment during exercise (10). It has been shown to have positive effects on both the body and mind (11, 12).

Most older adults prefer to participate in aerobic dance classes in community centers. Owing to the COVID-19 pandemic in recent years, activities in parks are likely to promote the spread of the virus, causing older adults in the community to be unable to participate and have insufficient activity and reduced physical fitness. Therefore, some scholars have advocated for the use of aerobic exercise videos to guide older adults to exercise at home and avoid a decline in physical fitness. However, the use of only aerobic exercise videos often leads to tendon strain or other sports injuries in older adults due to incorrect postures, thereby greatly reducing the effectiveness of these exercises. In addition, following only aerobic exercise videos may lead to excessive exercise in older adults, which can increase the risk of myocardial infarction and sudden cardiac death (13, 14).

Kinect is equipped with focus tracking technology, and the base motor rotates as the focused object moves. Many studies have pointed out that a Kinect sensor is used in conjunction with aerobic exercises to evaluate their accuracy by capturing the user's body movements (15, 16). When older adults exercise at home, they need to use wearable heartrate monitors to monitor exercise intensity. Furthermore, it can increase their safety when exercising at home (17). In view of this, this research will integrate the Microsoft Kinect intervention system and wearable heartrate monitoring to develop an improved cardiopulmonary training system based on aerobic dance courses for pre-frail community-dwelling older adults. However, scientific evidence on the effects of aerobic dance course exercises using the Kinect interventional system on physical fitness among community-dwelling older adults with pre-frailty is still lacking. Therefore, this study aimed to compare the effects of the Kinect intervention system and traditional dance training on physical fitness.

## 2 Materials and methods

### 2.1 Study design

This study used an assessor-blinded experimental design. This study was approved by the Institutional Review Board of Kaohsiung Veterans General Hospital, Taiwan (IRB No.: KSVGH20-CT4-25). The trial was registered at http://www.ClinicalTrials.gov (NCT06216236). Written informed consent was obtained from the individuals for the publication of any potentially identifiable images or data included in this article.

### 2.2 Subjects

Subjects were recruited from a local community. The inclusion criteria were: (1) individuals aged over 60 years; (2) meeting the “pre-frailty” criteria based on the SOF frailty scale; (3) able to walk independently for at least 5 min, with or without a walking aid; and (4) medically stable in the past 6 months.

Participants were excluded if they had a diagnosed psychiatric disorder or psychiatric comorbidities according to DSM-5 criteria. Those with clinically diagnosed dementia or other severe cognitive impairments (Mini-Mental State Examination score <24), as well as individuals with neurological, orthopedic, or unstable medical conditions, were also excluded.

### 2.3 Randomization and blinding

After baseline measurements, participants were randomly assigned to either the experimental group (Kinect aerobic dance training) or the control group (aerobic dance training) in a 1:1 ratio. Block randomization with a block size of 4 was used. The allocation was carried out by independent researchers using a computer-generated random number sequence. Although the instructors supervising the exercise sessions were aware of group assignments, outcome assessors and participants in both groups remained blinded throughout the study.

### 2.4 Intervention

**Figure 1:**
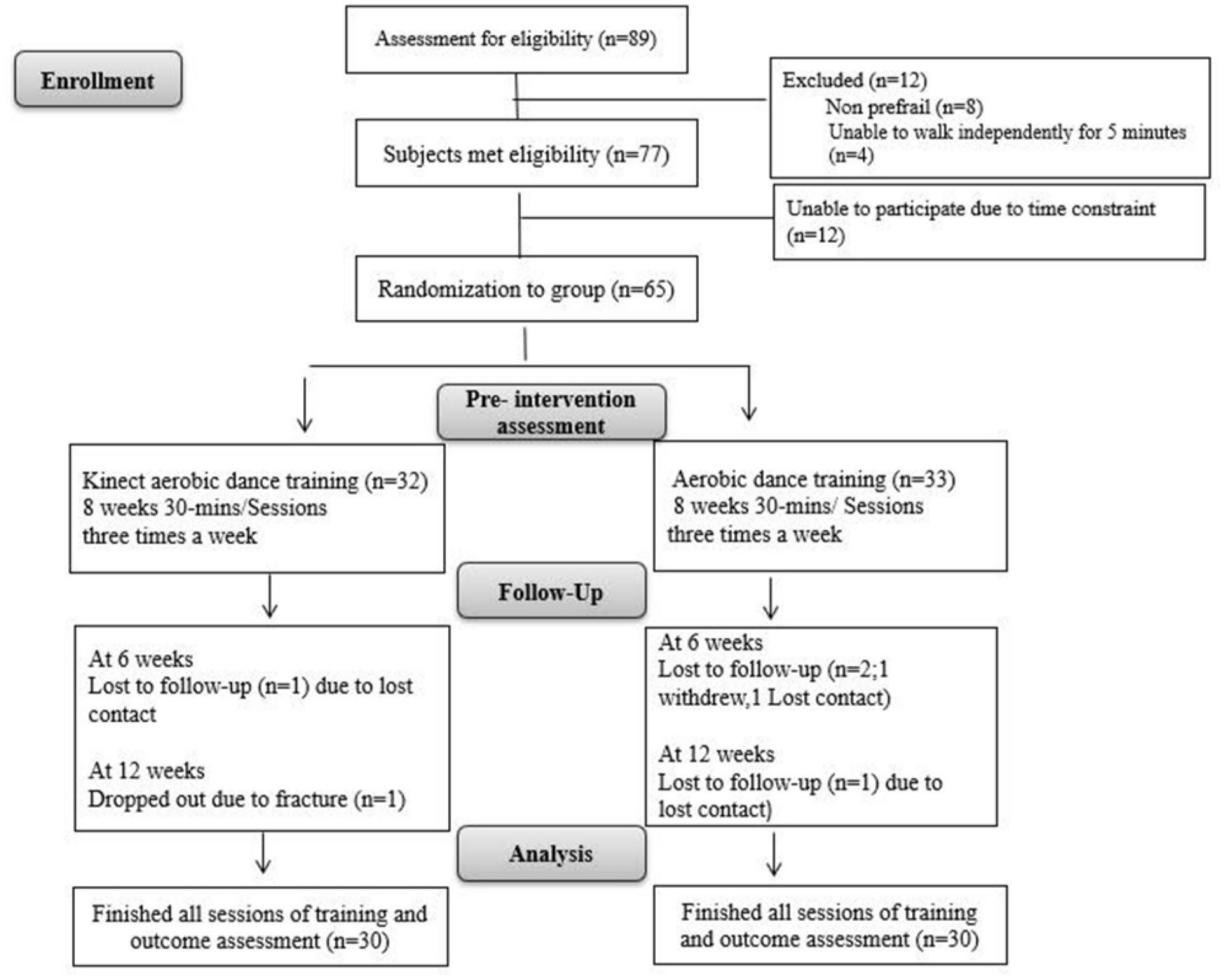
Flow chart of the study.

**Figure 2:**
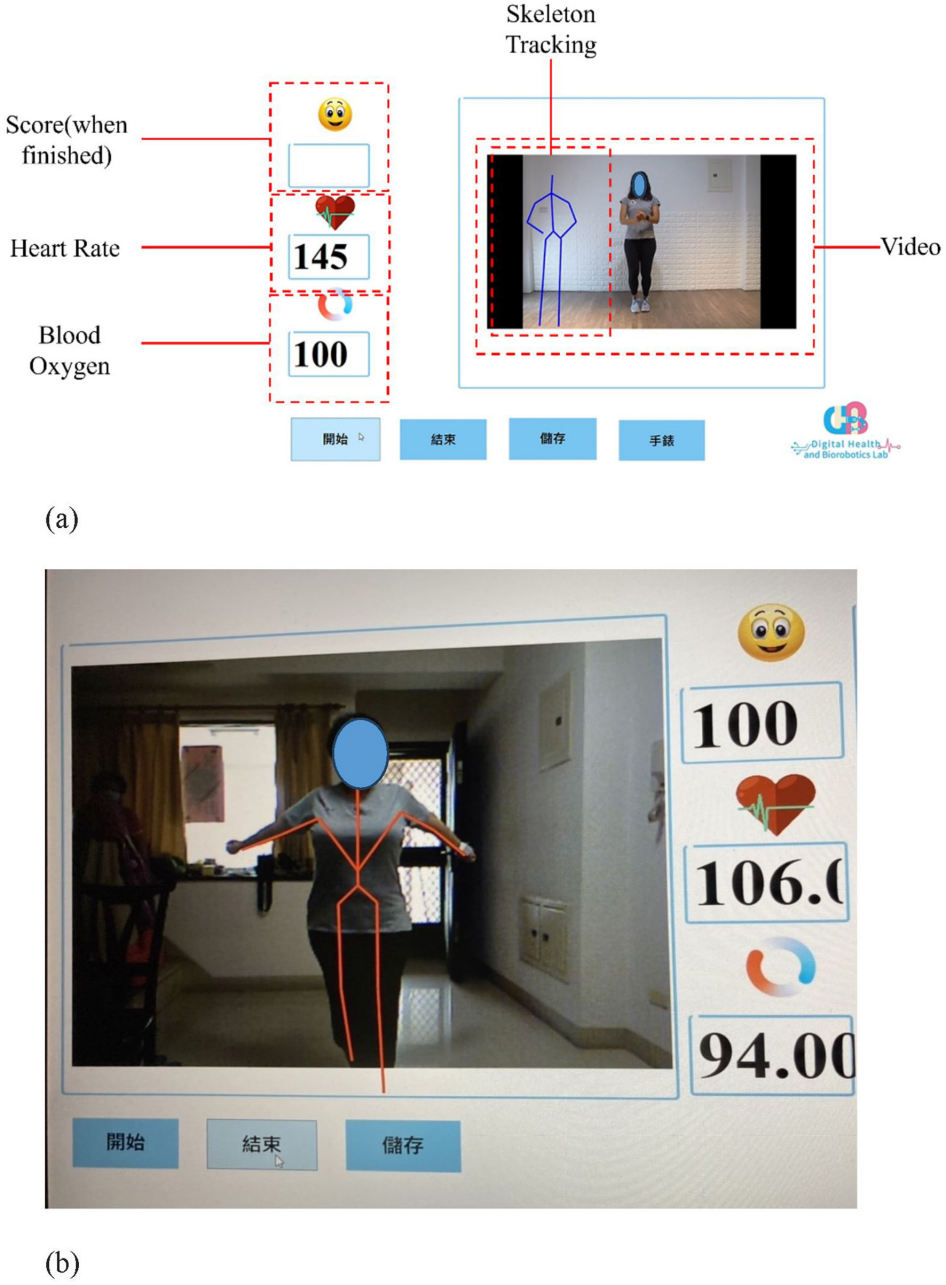
**(a)** An illustration of the Kinect system human-machine interface; **(b)** An illustration of the interface of the Kinect system while a participant was performing the aerobic dance exercise training.

The experimental group participated in aerobic dance sessions using the Kinect system. Each session lasted 30 min, and sessions were held three times a week for 8 weeks. The control group (*n* = 30) performed aerobic dance exercises at home. Outcome measures were assessed at three points: before training, after training, and 1 month after the training ended. A flowchart of the study design is shown in Figure 1. In this study, we developed an aerobic dance training program using the Kinect system. The system tracked joint positions and created a virtual skeleton to assess the accuracy of participants' movements. These movements were compared to those of a professional aerobic dance practitioner. If a participant's limb angles were within 5° of the standards, they earned points. No points were given if the movement was outside this range. The scoring system was weighted—more challenging movements earned more points. Participants could view their own skeletal motion projected on a screen alongside the professional model's movements. This allowed them to see how their performance compared to the ideal standard (Figure 2).

During the training session, the participants of the experimental group could see their own actions and those of a professional aerobic dance practitioner. The system would monitor and judge if the participant's action is correct for the whole duration of the video. The awarding score would display on the screen as an incentive for the participants. The control group used a video that only presented a professional aerobic dance practitioner's movement but did not show the skeletal simulation. There was also no performance score for feedback as incentives.

### 2.5 Outcome assessments

Assessment was performed at three time points: before the training period, right after the training period, and 1 month after. The primary outcome measures were physical fitness components: body mass index (BMI), waist-to-hip ratio (WHR), grip strength, 30 Second Chair Stand Test (30CST), and 6-minute walk (6MWT) tests (18). Since this exercise training focused on aerobic contents, flexibility was not assessed. The Quality-of-Life Questionnaire by World Health Organization (WHOQOL-BREF) (19) and Behavior Regulation in Exercise Questionnaire-2 (BREQ-2) (20) were also assessed to measure the quality of life and exercise behavior regulation and exercise enthusiasm.

#### 2.5.1 Body composition

Body mass index (BMI): the formula for calculating BMI is weight (kilograms, kg) divided by height squared (meters, m^2^). When the BMI index is lower than 18.5, it means underweight, 18.5–23.9 normal, 24–27 overweight, 28–32 obese, and higher than 32 very obese. When BMI reaches or exceeds 24, the probability of suffering from metabolic diseases, such as high blood pressure, diabetes, dyslipidemia, and other serious health-threatening diseases increases significantly.

Waist circumference and waist-to-hip ratio are important indicators of obesity in older adults, and are also predictors of simple cardiovascular diseases. Waist measurement is taken by standing with feet shoulder-width apart using a tape measure to measure the horizontal circumference parallel to the navel; hip circumference is the horizontal circumference of the widest hip. Waist-to-hip ratio is the ratio of waist circumference divided by hip circumference.

#### 2.5.2 Physical fitness assessment

##### 2.5.2.1 Grip strength

This is mainly to measure the muscle strength of the upper limbs. The subject used the dominant hand to bend the elbow at 90° in the standing position, and was asked to squeeze the Jamar manual ergometer (Jamar, Jackson, USA) with maximum strength; the measurement was repeated three times, with the rest time between repetitions being about 15 s; the three highest scores were recorded as the final score.

##### 2.5.2.2 30-Second Chair Stand Test

This is mainly used to measure the muscle endurance (strength/endurance) of the lower body. A 17-inches-wide chair was used with the patient's arms folded over the chest. The number of sit-to-stand repetitions completed over a 30-s period was recorded.

##### 2.5.2.3 Six-minute walk test

Used to assess the functional cardiorespiratory fitness (Cardiorespiratory endurance) (21) and mobility (mobility) of each subject. In older adults over 65 years old, the 6-min walk test has good reliability and validity, and the intraclass correlation coefficient is 0.91–0.96. Subjects were asked to measure the distance they walked (not running or jogging) in 6 min. During the 6MWT, subjects were directly observed by an evaluator, and the distance walked was recorded. While walking, a portable pulse oximeter was used to continuously monitor heart rate, respiratory rate, and blood oxygen concentration.

#### 2.5.3 Quality of life

The “Taiwan Concise World Health Organization Quality of Life Questionnaire” used in this study is a simplified version of the WHOQOL-100, developed by the WHOQOL-BREF group. The Chinese version of the WHOQOL-BREF includes one question from each of the 24 domains in the original WHOQOL-100. It contains 26 questions across four domains: physical health, psychological health, social relationships, and environment. In addition, the Taiwanese version includes two culturally relevant topics: “respect and acceptance (face and relationship)” and “food,” bringing the total to 28 items. Each question is rated on a five-point Likert scale. Scores for each domain range from 4 to 20, with higher scores indicating a better quality of life. The questionnaire has good internal consistency, with a reliability coefficient of 0.91. Its content validity ranges from 0.53 to 0.78. Participants completed the questionnaire on their own before taking the walking test.

#### 2.5.4 Behavior regulation in exercise questionnaire-2

The Chinese version of the Exercise Behavior Regulation Questionnaire is a measurement tool used to measure the general public's exercise motivation. The scale is divided into five aspects: internal regulation aspect, identity regulation aspect, interjective regulation aspect, external regulation aspect, and motivation (22). Cronbach's scale ranged from 0.71 to 0.91 with good internal consistency. The questionnaire was filled by the subjects themselves before the walking test.

### 2.6 Statistical analysis

The sample size was calculated using G^*^power software (Ver. 3.1), yielding an estimated sample size of 30 participants per group (power = 0.9, alpha = 0.05, effect size = 0.35). All statistical analyses were performed using SPSS software (IBM Corp, Released 2020). The descriptive statistics were provided as means with standard deviations for continuous variables, and as absolute and relative frequencies and percentages for categorical variables. Independent *t*-tests and chi-square tests were performed to compare baseline characteristics between the experimental and control groups.

Generalized estimating equation procedure, which extends the generalized linear model for the analysis of repeated outcome measurements was used to examine the effect between groups and times. Statistical significance was set at *p* < 0.05.

## 3 Results

### 3.1 Participants' basic information

Figure 1 showed the study flow. Initially 65 older adults participated in the study and randomly assigned to the experimental group or control group. But finally 60 participants finished the training session and all the measurements. Table 1 presents the basic characteristics of the participants. No significant differences of the basic characteristics or baseline measurements were observed between groups. The comparisons of the physical fitness outcome measures at weeks 0, 8, and 12 within groups and between groups were presented in Table 2.

**Table 1:** Descriptive characteristics of the pre-frail community-dwelling older adults.

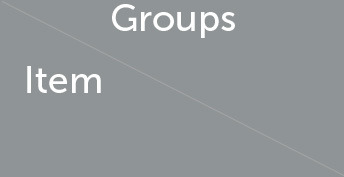	**Kinect intervention group (*N* = 30)**	**Control group (*N* = 30)**	***p*-Value**
Age (year)	69.10 ± 5.76	68.70 ± 4.31	0.762
**Sex**			
Female	25 (83.3%)	24 (80.0%)	0.111
Male	5 (16.7%)	6 (20.0%)	
Height (cm)	156.73 ± 7.15	157.80 ± 6.69	0.563
Weight (kg)	58.26 ± 9.25	60.27 ± 12.55	0.484
Number of falls (time/year)	0.37 ± 0.76	0.15 ± 0.40	0.176
BMI	23.78 ± 3.24	24.06 ± 4.60	0.789
**Education level**			
High school or above	19 (63.3%)	15 (50.0%)	0.297
Under junior high school	11 (36.7%)	15 (50.0%)	
**Living status**			
Live with family	26 (86.7%)	28 (93.3%)	0.671
Living alone	4 (13.3%)	2 (6.7%)	

Data are presented as mean ± SD for continuous variables; number (frequency) for category variables.

**Table 2:** Outcome measure scores between groups at week 0, 8, and 12.

**Weeks**	**Week 0**	**Week 8**	**Week 12**	**Week 0–8**	**Week 0–12 P^a^**	**Week 0–8 P^b^**	**Week 0–12 P^b^**
**BMI**							
EG	23.78 ± 3.24	23.41 ± 3.45	22.85 ± 5.12	0.057	0.205	0.309	0.214
CG	24.06 ± 4.60	24.02 ± 4.63	24.03 ± 4.65	0.326	0.231		
**WHR**							
EG	0.88 ± 0.06	0.88 ± 0.05	0.89 ± 0.06	0.105	0.225	0.503	0.268
CG	0.88 ± 0.07	0.88 ± 0.06	0.88 ± 0.06	0.592	0.797		
**30CST**							
EG	10.73 ± 3.02	13.13 ± 2.86	13.20 ± 3.01	<0.001^c^	<0.001^c^	0.002	0.277
CG	9.93 ± 2.63	10.63 ± 2.73	11.60 ± 3.07	0.038	<0.001		
**Grip strength**							
**Right hand**							
EG	22.43 ± 11.62	21.27 ± 8.22	21.00 ± 8.62	0.531	0.441	0.382	0.647
CG	20.53 ± 7.13	21.10 ± 7.42	20.03 ± 7.30	0.498	0.36		
**Left hand**							
EG	20.40 ± 7.47	21.10 ± 6.92	19.90 ± 7.66	0.186	0.485	0.723	0.373
CG	21.07 ± 7.60	21.47 ± 7.31	21.47 ± 7.31	0.566	0.596		
**6MWT**							
EG	359.07 ± 63.34	413.50 ± 95.46	431.53 ± 93.27	<0.001^c^	<0.001^c^	0.104	0.016
CG	363.90 ± 70.20	393.60 ± 71.56	397.63 ± 78.94	0.018	0.008		
**WHOQOL-BREF**							
EG	3.61 ± 0.37	3.85 ± 0.30	3.82 ± 0.31	<0.001^c^	0.003^c^	0.024	0.038
CG	3.64 ± 0.34	3.72 ± 0.37	3.67 ± 0.40	0.180	0.586		
**BREQ-2**							
EG	47.57 ± 6.75	49.87 ± 8.62	51.27 ± 5.56	0.180	0.008	0.936	1.000
CG	53.97 ± 17.22	55.97 ± 14.77	57.67 ± 14.09	0.560	0.279		

EG, experimental group; CG, control group; BMI, body mass index; WHR, waist to hip ratio; 30CST, 30 Second Chair Stand Test; 6MWT, 6-minute walk test; WHOQOL-BREF, Quality-of-Life Questionnaire by World Health Organization-BREF; BREQ-2, Behavior Regulation in Exercise Questionnaire-2.

^a^Intergroup analysis.

^b^Intragroup analysis.

^c^*p* < 0.05.

### 3.2 Changes in participants' physical fitness components

There were no significant changes and differences of BMI, WHR and grip strength within and both groups. Regarding the 30CST, both the experimental and control groups showed improvement during weeks 0–8 (*p* < 0.001) and 0–12 (*p* < 0.001). There was a significant difference between the two groups at 0–8 weeks (*p* < 0.002). Both the experimental and control groups improved 6MWT during weeks 0–8 (*p* < 0.001) and 0–12 (*p* < 0.001), and there was a significant difference between groups at 0–12 weeks (*p* < 0.016).

Regarding the secondary outcome measures (subjective measures), the experimental group showed improvement on the WHOQOL-BREF during weeks 0–8 (*p* < 0.001) and 0–12 (*p* < 0.003). No changes in the control groups. Therefore, a significant difference between groups at 0–8 weeks (*p* < 0.024) and 0–12 weeks (*p* < 0.038). There was an also significant difference in BREQ-2 scores in the experimental group at 0–12 weeks (*p* < 0.008), but no changes in the control group.

## 4 Discussion

Overall, both the experimental group and control group showed significant improvements in physical fitness and quality of life, as measured with the chair stand test and 6MWT. No improvements were observed in BMI, WHR, and grip strength.

A recent study has reported that using virtual reality (VR)-based training could reduce the risk of falls by improving static and dynamic balance (18). However, study report of the effect on physical fitness are limited. Our study showed a statistically significant improvement in chair stand test scores after the training sessions in both the experimental and control groups at 0–8 weeks. Previous research indicates that using a 360 Kinect of VR can improve lower body strength (18). The training in this study involved several lower limb activities, and aerobic exercise training was recreational for older adults. The amount of exercise did not exceed the muscle strength load, which may be why upper limb muscle strength did not increase.

The 6MWT results revealed considerable improvements in the pre-frail older adults community. The 6MWT is related to aerobic exercise. Therefore, community-dwelling older adults with pre-frailty were able to improve their cardiopulmonary fitness. A possible reason for the nonsignificant changes in the 6MWT in the control group was that their exercise load did not meet the required standards. Zeng et al. provided evidence that using the Xbox 360 Kinect Sensor with aerobic dance training had a positive effect on the physical state of older adults (19). The Kinect system can also capture participants' whole-body movements to help assess the correctness of their movements. The Kinect system converts a detected 3D image into a skeleton tracking system and can visually present the angles of body joints, allowing participants to perform aerobic exercises at home, which can maximize the effects of physical fitness training (20).

After 8 weeks of training, significant improvements in WHOQOL-BREF scores were observed. Quality of life is a measure of a measure of whether individuals can achieve spiritual and cultural pursuits (23). The virtual interactive interface detects skeletal structural characteristics of the human body's bones. It is used to collect whole-body movements of the subject during aerobic exercise, transmit them to the computer for simulation, and project them on a large screen to increase visual feedback and movement during aerobic exercise. Improve the accuracy of action execution and reduce errors caused by Kinect misjudgment during training (24).

Our study results also showed that there was a significant improvement in BREQ-2 scores. This indicates that motivation for exercise behavior is regulated. The subjects in the experimental group could see their performance and that of professional teachers to assess the correctness of their movements. Movement accuracy would be displayed when they completed the activities, increasing the motivation to perform activities (25, 26). For practical purpose, we recommend the exercise could be done in a community plaza or community square. Using this system could promote interaction with others and increases social participation.

## 5 Limitations and suggestion

This study had certain limitations. First, the exercise environments of the control and experimental groups were different, which might have affected participants' motivations. Second, this study involved only 4 weeks of follow-up; therefore, future studies with long-term follow-ups are needed to determine whether changes are long lasting.

## 6 Conclusion

In conclusion, both the experimental group and control group show improvement in participants' fitness in 30CST and 6MWT. However, the participants in the experimental group improved more. Moreover, they show significant improvement in quality of life, exercise behavior regulation and fitness enthusiasm. Thus, aerobic dance exercise using Kinect system was more effective than traditional training of aerobic dance exercise using video for pre-frail community-dwelling older adults.

## Data Availability

The original contributions presented in the study are included in the article/supplementary material, further inquiries can be directed to the corresponding authors.
